# Rupture of the Urethra, Etc.

**Published:** 1881-05-20

**Authors:** Thos. R. Wright

**Affiliations:** Georgia; Demonstrator of Anatomy Medical Department University of Georgia, at Augusta


### RUPTURE OF THE URETHRA WITH EXTRAVASATION OF URINE, ETC.

BY THOS. R. WRIGHT, M. D., DEMONSTRATOR OF ANATOMY MEDICAL DEPARTMENT UNIVERSITY OF GEORGIA, AT AUGUSTA.

Read before the "Augusta Academy of Medicine," March 2d, 1881.

Among the multitude of surgical troubles to which human flesh is heir, there is possibly none which causes the surgeon more anxiety and trouble than one of extensive urinary infiltration; and especially one in which the perinæum and scrotum have been infiltrated and distended with offensive decomposed urine. The following case, illustrative of this condition, may be of interest:

On the 22d of November, 1880, I was sent for to see Dennis A., a negro man about fifty years of age, the messenger stating "that the old man could not pass his water, and that what he did make went into his bag, which was as large as a baby's head." Upon seeing the patient, the following history was obtained: Some years before he had trouble in making water, and was then treated by a doctor who relieved him, and he had no more trouble until ten days before he sent for me, when he again had some difficulty in voiding his urine.

Thinking this was a small matter, he took some domestic remedies, hoping to get relieved, but failing with these, by the advice of friends, he partook very freely of watermelon-seed tea. After taking this tea his desire to pass water was so great he thought if he would strain with all his might he would be relieved; he did so, and making a violent effort he said he felt something give way, and was relieved for a while. Shortly after this, his privates began to swell and burn and pain him. On examination the perinæum, scrotum and lower portion of the abdominal wall were found infiltrated with urine, his scrotum, in fact, being "as large as a baby's head." From the history and condition of the patient I suspected the trouble, and tried to pass a No. 7 elastic catheter, but failed, the instrument meeting with what seemed, and afterwards proved to be, a very tight stricture a little anterior to the bulbous portion of the urethra. An attempt was then made to pass a filiform bougie, which was finally successful. After the bougie passed the stricture it entered any number of false passages, making it appear as though the urethra back of the stricture had been torn up by the rough use of instruments, and it was only after long and patient trial that the instrument was carried into the bladder. An attempt was then made to pass a larger instrument, but was unsuccessful. Not having my dilator or urethratome with me, I decided to incise his scrotum freely, put him thoroughly under quinine, and leave him until next morning, it being late in the afternoon when I saw him and some distance from the city.

Calling the next morning, with Prof. DeS. Ford, whom I had asked to see the case, we again tried to pass a large bougie, but failed. Ether was then given and another attempt made only to fail. The filiform guide was then passed, and upon it Gouley's tunnelled dilator and urethratome was carried through the stricture, when it was dilated and then cut. This was then withdrawn and a Gross' dilator introduced and the stricture dilated to 25 on the scale. A catheter was then easily carried into the bladder, a little ammoniacal urine flowing out, the instrument being left in the bladder. The scrotum had diminished some but was still very large, and was again incised. Five grains of quinine were ordered given every four hours with 15 drops of tinct. ferri chlor. three times a day, with brandy, milk and eggs.

On the third day my patient's general condition was a little better; he had removed the catheter, however, and his scrotum was larger than ever. His wife stated that he pulled the instrument out, and then desiring to urinate had gotten up to do so, and on making the effort "his privates (as she expressed it) swelled right out," no urine coming by the urethra. A catheter was again introduced into his bladder, and the necessity of its remaining there explained to him, the same directions for quinine, iron and nourishing diet being continued.

On the fourth day (25th) the man's condition was not so good; the scrotum and perinæal tissues in about the same condition, with the catheter again removed, no urine having passed since its withdrawal in the early part of the night. The condition of the man was now such that I began to think of the propriety of performing perinæal section; the man could only void his urine by the aid of the catheter, and this he would not allow to remain in his bladder, and any attempt to pass his water without it seemed only to force the urine through the ruptured urethra into the already distended scrotum and perinæum, and even while the catheter was in the bladder, there was no appreciable change in the infiltrated parts, thus leading me to believe the urine was passing along the outside of the instrument and getting into the tissues. I thought if the section was made and the urethra entered behind the seat of rupture and free passage given to the flow of urine, it might be of service to him, although I feared the operation had already been too long delayed. The operation, with its possible termination, was then explained to him and his family, when all agreed that it should be done. Accordingly the operation was decided upon, and with the assistance of Drs. DeS. Ford and Perrin, performed as follows: The patient being in the lithotomy position, a small grooved staff was introduced into the bladder and an incision made in the median perinæal line down upon the groove, and the urethra entered a little anterior to its bulbous portion. A female catheter was then passed through the perinæal opening into the bladder, a little urine flowing out. This instrument was withdrawn, and there being no blood the wound was left entirely open. In passing the finger into the wound it entered the false passages spoken of before, and could be passed through these into different parts of the perinæum and down into the scrotal tissues; the parts, to the touch, felt very hard and indurated, and cut like cartilage. The patient reacted from the operation very well; a ¼ grain sulph. morphia was given to relieve pain, with quinine, iron and nourishing diet, as before.

At my evening visit he expressed himself as being very comfortable, had suffered very little pain, and had voided his urine several times through the wound in the perinæum. The next morning after the operation his condition seemed very favorable, he had passed a comfortable night, had relished his breakfast, and felt better every way. The scrotum had diminished considerably in size, and he was passing his urine freely through the perinæal opening. At this visit, from my patient's general condition, I felt very much encouraged as to a favorable result, and hoped he might finally get well. But my visit next morning banished all my hopes, for a decided change for the worse was evident; his pulse was very feeble and frequent, with appetite gone, and he was lying in a semi-unconscious state. Brandy and milk was given him very freely, but to no avail; he gradually grew worse and died the next day, about 72 hours after the operation.

The treatment of this case is that which I believe is given by nearly all authorities upon the subject, *i. e.*, to give free passage to the flow of urine, incise freely the infiltrated tissues and support the system by tonics and stimulants, all of which was done in this case before the operation of perinæal section was resorted to, and it was only after seeing these measures fail that the operation was thought of. I believe now had the operation been performed at first the chances for my patient would have been better, for the strong constitution which he seemed to possess might then have enabled him to overcome the trouble. Indeed, should another case of the kind fall into my hands, I shall adopt the treatment so emphatically laid down by Prof. Van Buren, to perform external perinæal urethrotomy at once, and thus insure a free passage to the out-flow of urine, leaving the stricture to be treated at a subsequent time.

